# Correction for false statement

**DOI:** 10.1186/1687-9856-2012-1

**Published:** 2012-01-27

**Authors:** Milton Diamond

## 

Dear Editor,

A false statement has been published in your journal in an article by P. A. Lee and C. P. Houk. (2010) Article ID 563640. "The Role of Support Groups, Advocacy Groups, and Other Interested Parties in Improving the Care of Patients with Congenital Adrenal Hyperplasia: Pleas and Warnings [1]."

These authors say "Confrontational tactics used by "advocacy" groups have included pressuring the medical community to adopt narrow mandates, such as a moratorium on all reproductive system surgery (six), that cannot apply to the broad range of situations encountered in practice or the use of accusations regarding therapy received by patients in the past."

In support of their argument they cite our paper (M. Diamond & H. K. Sigmundson, Management of intersexuality. Guidelines for dealing with persons with ambiguous genitalia. *Archives of Pediatrics & Adolescent Medicine*. 1997;151(10):1046-1050 [2]. We have been wrongly quoted and cited.

Here is exactly what we say:

"Perform no major surgery for cosmetic reasons alone; only for conditions related to physical/medical health. This will entail a great deal of explanation needed for the parents who will want their children to "look normal." Explain to them that appearances during childhood, while not typical of other children, may be of less importance than functionality and post pubertal erotic sensitivity of the genitalia. Surgery can potentially impair sexual/erotic function. Therefore such surgery, which includes all clitoral surgery and any sex reassignment, should typically wait until puberty or after when the patient is able to give truly informed consent." [emphasis added].

Our emphasis was on cosmetic surgery and since our statement no evidence has shown *any *surgery had been of benefit. Many unneeded surgeries have been devastating.

Significantly damaging were those surgeries imposed on males that were sex reassigned for different reasons. Often these were instances where the penis was considered too small for appropriate male status; e.g., the John/Joan case [3,4] but there now have been many instances recorded where males were reassigned and raised as girls and then rebelled to live as males; particularly for cases of cloacal exstrophy [5,6]. Cases of micropenis are also currently recognized as not needing surgery or sex reassignment [7,8].

Creighton (2004), for example, has found "in girls with ambiguous genitalia, vaginoplasty is commonly performed during the first year of life . . . although the child is unlikely to be sexually active until after puberty. There is no good evidence it is justified. [9]" And Alizai et al. (1999) reported, "The outcome of clitoral surgery was unsatisfactory (clitoral atrophy or prominent glans) in [girls] whose genitoplasty had been performed by 3 different specialist pediatric urologists. Additional vaginal surgery was necessary for normal comfortable intercourse in [other] patients. Fibrosis and scarring were most evident in those who had undergone aggressive attempts at vaginal reconstruction in infancy [10]." Minto and others [11,12] echo similar expressions against early genital surgery. Schober has stated "A reliable, successful genitoplasty procedure that can be performed early in childhood for either feminization or masculinization has not yet been developed [13]." Legal and ethical reasons against such cosmetic surgery on infants have been presented [14] and argued against [15].

## References

[B1] Lee PA Houk CP The Role of Support Groups, Advocacy Groups, and Other Interested Parties in Improving the Care of Patients with Congenital Adrenal Hyperplasia: Pleas and Warnings International Journal Paediatric Endocrinology 2010 10.1155/2010/563640 PMC2905711 20652077

[B2] Diamond M Sigmundson HK Management of intersexuality. Guidelines for dealing with persons with ambiguous genitalia Archives of Pediatrics & Adolescent Medicine 1997 151 10 1046 1050 10.1001/archpedi.1997.02170470080015 9343018

[B3] Colapinto J As nature made him: The boy who was raised as a girl 2000 Harper Collings, New York

[B4] Diamond M Sigmundson HK Sex Reassignment at Birth: Long Term Review and Clinical Implications Archives of Pediatrics and Adolescent Medicine 1997 151 298 304 10.1001/archpedi.1997.02170400084015 9080940

[B5] Reiner WG Gender Identity And Sex Assignment: A Reappraisal For The 21st Century 2002 10.1007/978-1-4615-0621-8_11 12575762

[B6] Reiner WG Diamond M, Yates A Cloacal Exstrophy Child and Adolescent Psychiatric Clinics: Sex and Gender 2004 Elsevier, Philadelphia

[B7] Reilly JM Woodhouse CRJ Small penis and the male sexual role The Journal of Urology 1989 142 569 572 2746779 10.1016/s0022-5347(17)38818-3

[B8] Wisniewski A Migeon C Gender identity/role differentiation in adolescents affected by syndromes of abnormal sex differentiation Adolesc Med 2002 13 119 28 11841959

[B9] Creighton SM Sytsma SE Adult Outcomes of Feminizing Surgery Ethics and Intersex 2004 Springer, Dordrecht, The Netherlands 207 214

[B10] Alizai NK Thomas DF Lilford RJ Batchelor AG Johnson N Feminizing genitoplasty for congenital adrenal hyperplasia: What happens at puberty? Journal of Urology 1999 161 1588 1591 10.1016/S0022-5347(05)68986-0 10210421

[B11] Minto CL Creighton S Long term sexual function in Intersex Conditions with Ambiguous Genitalia Journal of Pediatric and Adolescent Gynecology 2002 14 141 142

[B12] Minto CL Liao L-M Woodhouse CRJ Ransley PG Creighton SM The effect of clitoral surgery on sexual outcome in individuals who have intersex conditions with "ambiguous genitalia" a cross-sectional study The Lancet 2003 361 1252 1257 10.1016/S0140-6736(03)12980-7 12699952

[B13] Schober JM Sytsma SE Ethics and Futuristic Scientific Developments Concerning Genitplasty Ethics and Intersex 2006 Springer, Dordrect 311 317

[B14] Beh HG Diamond M An emerging ethical and medical dilemma: Should physicians perform sex assignment surgery on infants with ambiguous genitalia? Michigan Journal of Gender and Law 2000 7 1 63 12715806

[B15] Diamond M Pediatric management of ambiguous and traumatized genitalia Journal of Urology 1999 162 1021 1028 10.1016/S0022-5347(01)68054-6 10458424

